# Different ECLS Pump Configurations for Temporary Right Ventricular Assist Device in LVAD Patients: A Retrospective Case–Control Study

**DOI:** 10.3390/life14101274

**Published:** 2024-10-07

**Authors:** Dragan Opacic, Christian Klüß, Darko Radakovic, Georges El-Hachem, Tobias Becker, Markus Rudloff, Volker Lauenroth, Marcus-André Deutsch, Claudio Velasquez-Silva, Henrik Fox, René Schramm, Michiel Morshuis, Jan F. Gummert, Sebastian V. Rojas

**Affiliations:** 1 Clinic for Thoracic and Cardiovascular Surgery, Heart and Diabetes Centre North Rhine Westphalia, 32545 Bad Oeynhausen, Germany; 2 Faculty of Medicine, Ruhr University Bochum, 44801 Bochum, Germany; 3 Medical Faculty OWL, Bielefeld University, 33604 Bielefeld, Germany

**Keywords:** acute right ventricular failure, left ventricular assist device, paracorporeal right ventricular assist device, extracorporeal life support pump configurations, bleeding

## Abstract

Background: Acute right ventricular failure is a critical complication after left ventricular assist device (LVAD) implantation, often managed with a temporary paracorporeal right ventricular assist device (RVAD). This study examined three extracorporeal life support (ECLS) systems regarding mortality, bleeding complications, and intensive care unit (ICU) stay duration. Methods: This monocentric, retrospective case–control study included all patients receiving LVAD with paracorporeal RVAD between 2009 and 2020. Three patient groups were formed: Centrimag^TM^ (A), Cardiohelp^TM^ (B), and Deltastream^TM^ (C). Results: A total of 245 patients were included. Preoperative parameters were similar between the Centrimag^TM^ and Deltastream^TM^ groups, but Cardiohelp^TM^ patients had worse Interagency Registry for Mechanically Assisted Circulatory Support (INTERMACS) Scores (A: 1.7 ± 0.8, B: 1.36 ± 0.5, C: 1.9 ± 0.9; *p* < 0.05). In-hospital death rates were A: 61 (41.8%), B: 15 (32.6%), C: 29 (54.7%); *p* < 0.05, and reoperation due to bleeding rates were A: 32 (21.9%), B: 8 (17.4%), C: 25 (47.2%); *p* < 0.05, with the Deltastream^TM^ group showing the highest rates. This group also had increased thrombocyte consumption and prolonged ICU stays. Conclusions: Temporary RVADs lead to bleeding complications, affecting patient outcomes. The Deltastream^TM^ group had significantly higher bleeding complications, likely due to high pump revolution rates and thrombocyte decline. Due to the study’s retrospective nature and complex patient profiles, these interesting findings should be validated in future studies.

## 1. Introduction

Heart failure is a growing public health problem that affects millions of people worldwide. In recent years, left ventricular assist device (LVAD) therapy has emerged as an effective treatment option for patients with end-stage heart failure who are not candidates for heart transplantation or have not responded to conventional medical therapy [1,2,3,4]. While LVADs can significantly improve the quality of life and survival of these patients, they are not without complications. One of the most dreaded complications of LVAD implantation is acute right ventricular failure (RVF), which occurs in up to 40% of cases [5,6,7,8,9].

RVF is associated with significant morbidity and mortality and can be caused by a variety of factors, including elevated pulmonary vascular resistance, RV ischemia, and mechanical factors related to LVAD implantation [8,10]. To treat this condition, the most established approach is the implantation of a temporary paracorporeal RVAD [11,12,13]. The temporary RVAD can be used as a bridge to recovery or as a bridge to a permanent RVAD or transplantation [11,12].

Extracorporeal life support (ECLS) systems are commonly used for temporary RVAD implantation in LVAD patients. ECLS systems consist of a pump, oxygenator, and cannulas that are connected to the patient’s vascular system to provide circulatory and/or respiratory support [13,14,15]. Several different ECLS pump configurations are available, each with its own advantages and disadvantages. The most commonly used ECLS pumps for temporary RVAD implantation include the Centrimag^TM^ (Abbott, Abbot Park, IL, USA), the Cardiohelp^TM^ (Maquet, Getinge Group, Rastatt, Germany), and the Deltastream^TM^ (Xenios AG, Fresenius Medical Care, Heilbronn, Germany).

Several studies have reported the effectiveness of temporary RVAD implantation in treating RVF following LVAD implantation [12,13,14,15]. However, there is still no consensus on the optimal ECLS pump configuration for temporary RVAD implantation. Despite the widespread use of ECLS systems for temporary RVAD implantation, little is known about the differences in outcomes between the various pump configurations.

Therefore, in this study, we aimed to investigate the differences between these three systems in terms of mortality, bleeding complications, and postoperative length of stay in the intensive care unit (ICU) in LVAD patients with acute RVF. The findings of this study could help guide clinicians in selecting the most appropriate ECLS pump configuration for their patients, potentially reducing the incidence of complications and improving outcomes.

## 2. Materials and Methods

### 2.1. Ethical Statement

The ethics committee of Ruhr University—Bochum, Germany, granted approval for the study (AZ: 2021-752). A written informed consent was not deemed necessary.

### 2.2. Study Design and Data Collection

**Figure 1:**
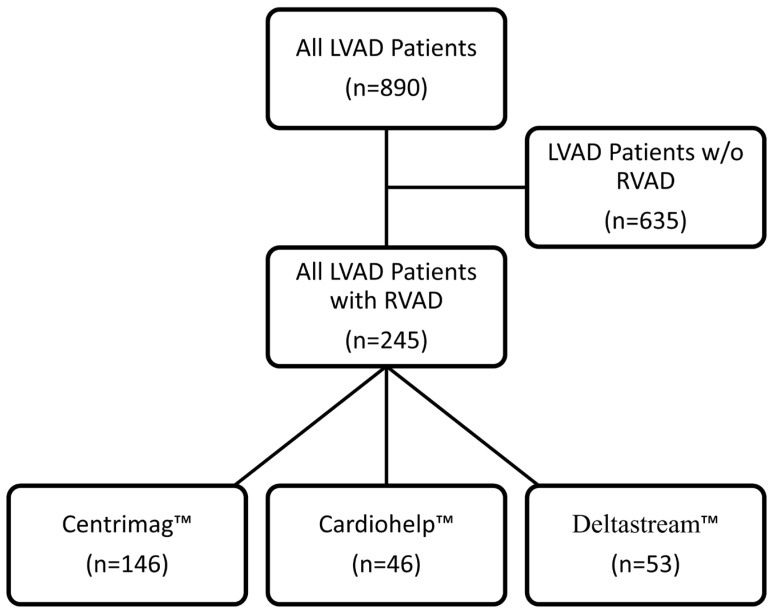
Study design.

To realise this study, we have performed a retrospective data analysis of all patients who received an LVAD system and a temporary RVAD for right ventricular failure (RVF) between 2009 and 2020. All patients in our study had either HVAD^TM^ (HVAD, Medtronic, Dublin, Ireland) or HeartMate3^TM^ (HM3, Abbott, Abbot Park, IL, USA) LVAD systems implanted. Patients who developed RVF received one of three ECLS systems regularly used in our clinic. The systems are Centrimag™, Cardiohelp™, and Deltastream^TM^, and their use was used based on the availability at the moment. The study design and the patient allocation are presented in Figure 1.

The data were retrieved from the prospectively kept database and electronic patient records. Long term survival was assessed with a regular review of medical records and repeated contacts with the patients as well as from the national register for birth and deaths.

### 2.3. RVAD Implantation and Weaning Protocol

In the case of the RVF, a temporary paracorporeal percutaneous RVAD was implanted. The diagnosis of RVF and the decision to implant temporary RVAD was made as a joint decision between the surgeon, anaesthetist, VAD coordinator, and perfusionist. The parameters relevant to the decision were cardiac output, central venous pressure, blood pressure, contractility assessed using transoesophageal echocardiography, and failure to achieve sufficient calculated cardiac output using the LVAD.

Once the decision was made, all patients were supported with percutaneous RVAD implanted in the usual manner. The inflow cannula (21 or 23 Fr, Getinge Group, Rastatt, Germany) was inserted percutaneously into the vena femoral using the Seldinger technique with the cannula tip just below the right atrium. An 8 mm Dacron prosthesis (Getinge Group, Rastatt, Germany) was anastomosed to the pulmonary artery with a 5-0 Prolene and exteriorised subxiphoidaly. The Dacron prosthesis was then cannulated with a 22 Fr EOPA Outflow cannula (Medtronic, Dublin, Ireland). Once the cannulas were in place, they were connected to one of the three ECLS systems. All blood-bearing surfaces of the system were coated (phosphorylcholine- or heparin-coated) so that systemic anticoagulation could be minimal. Namely, once the patient showed no increased bleeding, systemic anticoagulation with heparin or argatroban (*int. Tirofiban*) in the case of heparin-induced thrombocytopenia was initiated 6 h after the surgery at the earliest. The target partial thromboplastin time for patients on heparin therapy was set between 50 and 60 s. Meanwhile, the dosing of argatroban (*int. Tirofiban*) was adjusted based on its direct levels in the blood, aiming for a target range of 0.2 to 0.4 µg/mL. Once bleeding was under control, an additional 100 mg of Aspirin daily was incorporated into the standard therapy.

The weaning of the RVAD started as soon as the LVAD flow stabilised and it was possible to reduce the catecholamines. After the catecholamines reduction, the decrease in RVAD flow followed. The threshold to wean the patient from the RVAD was an RVAD flow of about 1.5 L/min in the presence of sufficiently calculated cardiac output using the LVAD, adequate gas exchange parameters, and a regular size and contractility of the left and right ventricle, as well as the mid position of the interventricular septum in echocardiography. Simultaneously, if the lung function and gas exchange parameters were appropriate, the oxygenator function was reduced and, if possible, removed before complete weaning from RVAD.

To remove RVAD canulae, the outflow cannula was removed from the Dacron prosthesis, which was then ligated and resected beneath the skin level. The venous cannula was pulled out and compressed until haemostasis.

### 2.4. Endpoints

The primary endpoints were intra-hospital mortality and reoperation due to bleeding complications. The secondary endpoints were transfusion rates, ICU and total hospital stay, successful RVAD weaning, and heart transplant.

### 2.5. Statistical Analysis

The statistical analysis was performed with the IBM Statistical Package for the Social Sciences (SPSS) Version 24 (IBM Corp., Armonk, New York, NY, USA). Categorial variables are presented as counts and percentages and analysed using Pearson’s Chi-square test, Yates’s correction and Fisher’s exact tests, as appropriate. Continuous parametric variables are presented as the mean and standard deviation, while nonparametric continuous variables are presented as medians and interquartile range. As appropriate, continuous variables are analysed with ANOVA or Kruskal–Wallis. Bonferroni correction was used for post hoc analysis. In order to account for baseline differences between the groups, the multivariate linear and logistic regression models were used to identify independent predictors for relevant study endpoints. Preoperative parameters with a *p*-value less than 0.200 were included in regression models and a conditional backward stepwise removal method was used to generate final regression model. Kaplan–Meier curves were used to compare survival over time between the groups. Statistical significance was considered at a *p*-value of less than 0.05.

## 3. Results

### 3.1. The Patient Characteristics

Our study included 245 patients that received LVAD and percutaneous temporary RVAD distributed in three groups based on the RVAD ECLS system used. The largest group was the Centrimag^TM^ group, with 146 patients, followed by the Deltastream^TM^ (*n* = 53) and Cardiohelp^TM^ (*n* = 46) groups. Figure 1. The patients in the Deltastream^TM^ group were older compared to the other two groups, but this difference was only statistically significant compared to the Cardiohelp^TM^ group (Centrimag^TM^ 55.5; 17 years, Cardiohelp^TM^ 52.5; 12 years, and Deltastream^TM^ 66.0; 11 years ^‡^, *p* = 0.039). The males were predominant in all three groups, ranging from 75 to 85%. The Cardiohelp^TM^ group had the lowest median Interagency Registry for Mechanically Assisted Circulatory Support (INTERMACS) score of 1; 0 compared to Centrimag^TM^ (2; 1) and Deltastream^TM^ (2; 2). A high INTERMACS Score in the Cardiohelp^TM^ group was in accordance with more prevalent preoperative MCS support, either with ECLS (56.5%, *p* = 0.001) and the use of Impella^TM^ (Abiomed, Danvers, MA, USA) (17.4%, *p* = 0.040) as well as preoperative cardiopulmonary resuscitation (15.2%, *p* = 0.14) in this group. Patients in the Deltastream^TM^ group had significantly more often undergone previous cardiac surgery (30.2%, *p* = 0.009) compared to both Centrimag^TM^ (17.1%) and Cardiohelp^TM^ (8.7%) group. See Table 1 for the patient characteristics.

Overall, ischemic cardiomyopathy (105 patients, 45.4%) and dilated cardiomyopathy (84 patients, 34.3%) were the most prevalent cardiomyopathy forms across all groups. Ischemic cardiomyopathy was more common in the Deltastream^TM^ group (56.6%) compared to the Centrimag^TM^ (37.0%) group. Dilated cardiomyopathy due to myocarditis was significantly more frequent in the Centrimag^TM^ group (21.2%) compared to both the Cardiohelp^TM^ (6.5%) and Deltastream^TM^ (3.8%) groups. Other cardiomyopathies and heart diseases showed no statistically significant differences between the groups.

The patients in the Cardiohelp^TM^ group were most often supported with a preoperative ECLS system, resulting in the LVAD implantation in many cases being conducted without conventional cardiopulmonary bypass. Cardiopulmonary bypass was, therefore, significantly more used in the Centrimag^TM^ group compared to Cardiohelp^TM^ (84.9% vs. 58.7%, *p* < 0.001). Consecutively, hemofiltration was the least used in the Cardiohelp^TM^ group compared to the Centrimag^TM^ and the Deltastream^TM^ group (15.2% vs. 52.5% vs. 35.8%, respectively, *p* = 0.004). Being a compact ECLS system with a fully integrated pump and oxygenator, all Cardiohelp^TM^ patients received an oxygenator, which was significantly more common compared to the Centrimag^TM^ and Deltastream^TM^ groups (100% vs. 69.2% 83.0%, respectively, *p* < 0.001). Additionally, in the Cardiohelp^TM^ group, somewhat more HVADs were implanted (Centrimag^TM^ 71.9% vs. Deltastream^TM^ 75.5%, Cardiohelp^TM^ 89.1%, *p* = 0.040).

All measured laboratory parameters varied highly between the patients, and therefore the data are presented as mean ± standard deviation and median and interquartile range. The most significant differences in laboratory parameters were observed in the liver enzymes (ALT, AST, LDH) that were significantly highest in the Cardiohelp^TM^ group; see Table 2. Additionally, patients in the Cardiohelp^TM^ group had significantly higher CK values and lower thrombocyte counts compared to the Centrimag^TM^ group.

### 3.2. The Study Endpoints

LVAD patients with acute RVF and RVAD have, unfortunately, atrocious prognosis. This was also observed in our study. The highest intra-hospital mortality was observed in the Deltastream^TM^ group with 54.7%. The intra-hospital mortality was significantly higher compared to Cardiohelp^TM^ (32.6%, *p* = 0.042) but not compared to the Centrimag^TM^ group (41.8%, *p* = 0.111). Similarly, reoperation due to bleeding was most common in the Deltastream^TM^ group, where 47.2% of patients underwent reoperation, which was significantly more common compared to both Centrimag^TM^ and Cardiohelp^TM^ groups (41.8%, *p* < 0.001 and 17.4%, *p* = 0.003, respectively). Interestingly, the total number of surgical revisions was slightly higher in the Deltastream^TM^ group, but this difference was not deemed significant due to sizeable interpatient variation; see Table 3.

Similarly, the Deltastream^TM^ group had the longest ICU stay (68 ± 70.5 days) and the longest time with invasive respiration (1069.1 ± 932.6 h), which was significantly longer only compared to the Centrimag^TM^ group (47.3 ± 41.4 days, *p* = 0.029 and 694.9 ± 732.4 h, *p* = 0.013, respectively). See Table 3.

In order to adjust for baseline differences between the groups, we have performed a multivariate binary logistic regression as described previously. The multivariate analysis showed that the independently relevant factors for intra-hospital mortality are age (OR 1.058, 95%CI 1.029–1.087, *p* < 0.001), previous cardiac surgery (OR 2.192, 95%CI 1.035–4.644, *p* = 0.040), and the use of Deltastream^TM^ ECLS system (OR 2.684, 95%CI 1.041–6.920, *p* = 0.041). A clinically not significant effect on intra-hospital mortality had preoperative serum LDH values (OR 1.000, 95%CI 1.029–1.087, *p* = 0.031), while the requirement for the oxygenator had a remarkable but statistically still not significant effect (OR 2.094, 95%CI 0.995–4.407, *p* = 0.051). See Table 4.

Furthermore, the multivariate analysis showed that the most relevant independent risk factor for reoperation due to bleeding was the use of Deltastream^TM^ ECLS system (OR 2.662, 95%CI 1.307–5.423, *p* = 0.007). The low preoperative Antithrombin III values were also a statistically significant risk factor for reoperation due to bleeding, but its effect was not so profound (OR 1.019, 95%CI 1.003–1.036, *p* = 0.021). See Table 4.

The higher rate of bleeding complications in the Deltastream^TM^ group was associated with higher transfusion volumes compared to the other two ECSL systems. This difference was the most substantial regarding thrombocytes transfusion. Namely, Deltastream^TM^ patients received 32.28 ± 27.72 thrombocytes transfusion units, which was significantly higher compared to both Centrimag^TM^ (20.0 ± 17.7 units, *p* < 0.001) and Cardiohelp^TM^ (17.9 ± 15.4, *p* < 0.001). Erythrocyte transfusion was also the greatest in the Deltastream^TM^ group (73.1 ± 47.3 units) compared to the other two ECLS systems (*p* = 0.012). Regarding the fresh frozen plasma transfusion, a significant difference was only observed between the Deltastream^TM^ and the Cardiohelp^TM^ group (30.8 ± 26.7 vs. 16.5 ± 17.5 units, *p* = 0.009). See Table 5.

The multivariate analysis showed that the amount of erythrocytes transfusion was strongly associated with the use of an oxygenator and the INTERMACS score. The strongest effect on the thrombocyte transfusion was found with Deltastream^TM^, while the Cardiohelp^TM^ system was associated with lower frozen fresh plasma transfusion. See Table 6.

**Figure 2:**
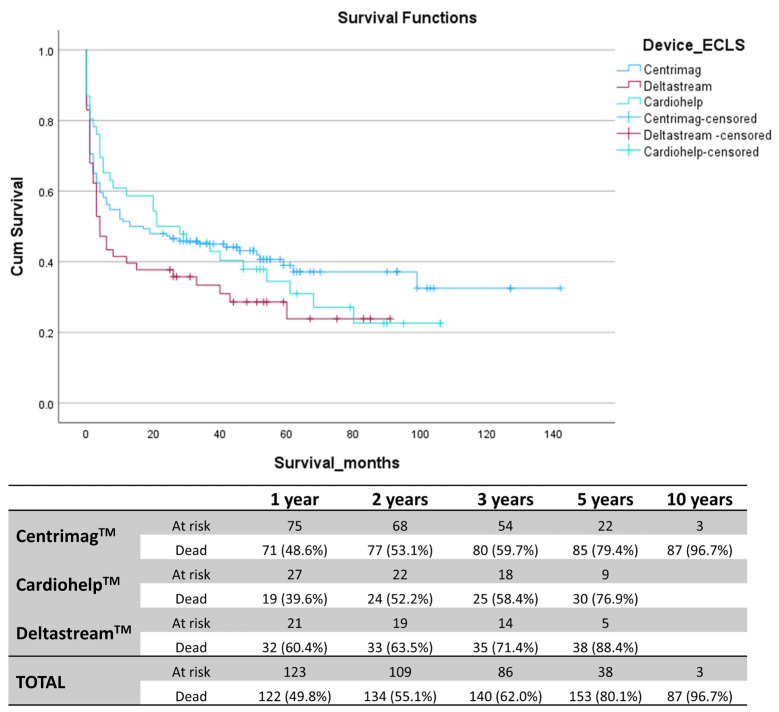
Kaplan–Meier survival curves for patients receiving LVAD and different temporary RVADs, Centrimag^TM^ (blue), Deltastream^TM^ (red), and Cardiohelp^TM^ (green). Table representing the total number of patients at risk and deceased patients at 1, 2, 3, 5, and 10 years following the LVAD implantation.

RVAD weaning was successful only in 13.2% of Deltastream^TM^ patients, which was significantly lower than both the Centrimag^TM^ (28.1%, *p* = 0.038) and the Cardiohelp^TM^ group (32.6%, *p* = 0.029). Similarly, only 9.4% of patients with the Deltastream^TM^ were transplanted, while the transplantation rate in the Centrimag^TM^ and Cardiohelp^TM^ group was significantly higher (24.7%, *p* = 0.018 and 26.1%, *p* = 0.035). Although the Deltastream^TM^ group initially had worse outcomes than other ECLS systems, this difference was diminished during the follow-up period. See Figure 2.

## 4. Discussion

The major finding of our study is that LVAD patients who develop acute RVF and have to be treated with temporary RVAD often suffer from bleeding complications, which results in numerous revisions and mass transfusions. One of the most influential risk factors for revision due to bleeding is using the Deltastream^TM^ ECLS system, which was associated with the highest intra-hospital mortality in these patients. The Deltastream^TM^ system is also associated with extreme thrombocyte transfusions, which can be the driving cause of haemorrhagic diathesis in these patients.

Studies in these highly complex patients are arduous to standardise and are often associated with significant baseline differences between the groups. The Cardiohelp^TM^ group had a significantly higher proportion of patients with INTERMACS profile 1 than the Centrimag^TM^ and Deltastream^TM^ groups, indicating worse outcomes [10,16]. This was reflected in the higher proportion of patients in the Cardiohelp^TM^ group who required preoperative mechanical support, either ECLS or Impella^TM^. ECLS implantation before surgery resulted in a significantly rarer use of classic cardiopulmonary bypass during LVAD implantation. In these patients, it is widespread and efficient to implant LVAD only under ECLS [17]. Furthermore, it is well established that the preoperative status of vital organs significantly impacts overall outcomes following LVAD implantation [18,19,20,21]. The laboratory findings showed that the Cardiohelp^TM^ group had a higher burden of renal and liver disfunction, which was in line with a low INTERMACS profile. These numerous differences in preoperative parameters could influence the study’s outcomes and should be considered when interpreting the results.

The Deltastream^TM^ group had significantly higher intra-hospital mortality compared to other groups. This observation was confirmed using multivariate analysis. Notably, previous cardiac surgery, more common in the Deltastream^TM^ group, was also defined as an independent risk factor for intra-hospital mortality. Almost half of the patients in the Deltastream^TM^ group underwent surgical revision due to bleeding. Multivariate analysis recognised the use of the Deltastream^TM^ system as an independent risk factor for the bleeding-associated revision. Angleitner P. et al. clearly showed that the bleeding revision has a detrimental effect on postoperative morbidity and mortality [22]. Therefore, it is clear that preventing bleeding complications should be one of the primary goals in the treatment strategies of these complex patients [23,24]. One of the most striking findings was that the Deltastream^TM^ group had an extremely high thrombocyte transfusion rate, signalling the potential driving mechanism behind the high incidence of bleeding complications in this group of patients. The intrinsic characteristic of the Deltastream^TM^ system that differentiates it from the other two is its significantly higher revolution rates. These high pump revolution rates could cause thrombocyte destruction and lead to bleeding complications.

The high reoperation rate in the Deltastream^TM^ group was accompanied by significantly higher erythrocyte and fresh frozen plasma transfusions. However, the Deltastream^TM^ system was not considered an independent risk factor for red blood cell transfusion, although the effect was quite considerable (B = 14.7, *p* = 0.057). When it comes to erythrocyte transfusion, the initial status of the patients, i.e., the INTERMACS score and the use of an oxygenator, had a more significant impact. Our data have shown that using an oxygenator had the most substantial impact on the erythrocyte transfusion rates. The oxygenator use significantly increases the contact surface of the ECLS system and, by that means, increases haemolysis [25]. Haemolysis seems to be a more relevant cause for haemoglobin decline and consecutive erythrocyte transfusions rather than bleeding, especially in patients with prolonged paracorporeal mechanical circulatory support [26]. The study by Lorusso et al. suggested that the choice of the ECLS system should be tailored to an individual patient’s needs and conditions [27]. This implies that the use of an oxygenator should be considered based on the specific requirements of each patient. However, it is common for the patient’s status to change over time. For example, some patients who initially require oxygenator support can recover from the lung perspective but still demand support for the failed right ventricle. In contrast, in others, lung function can decline over time, and they may require postponed lung support. Moreover, Boulate et al. highlighted that the implantation of a long-term MCS device in patients on ECLS could result in severe acute lung injury associated with ominous outcomes [28].

Patients who required hemofiltration in addition to LVAD and RVAD support demonstrated worse overall outcomes, including higher mortality rates and prolonged ICU stays [29]. Hemofiltration is typically required in patients with significant renal dysfunction, which is an indicator of multiorgan failure and severe hemodynamic instability. The need for hemofiltration reflects a more critically ill patient population, and its use likely contributed to an impaired prognosis [29]. Although in our dataset hemofiltration was not identified as an independent risk factor for mortality in the multivariate analysis, patients requiring this intervention were already at a higher risk due to underlying renal failure, which compounded their poor prognosis.

The type of LVAD received by patients did not significantly influence the overall findings of our study. Both HeartMate3™ and HVAD™ systems were used across the patient groups, and while there were minor variations in outcomes related to device-specific factors, these did not reach statistical significance in influencing the primary endpoints such as mortality, bleeding complications, or ICU stay duration. The allocation of the LVAD type was primarily determined by availability and institutional practice at the time of implantation, and both devices performed similarly in terms of hemodynamic support. Therefore, the higher bleeding rates and mortality observed in the Deltastream™ group were more closely associated with the RVAD system and its mechanical properties, such as high pump revolution rates, rather than the type of LVAD used.

With all that in mind, it is essential to evaluate the status of the patients and remove or add an oxygenator on time to secure adequate gas exchange in patients. From that perspective, the Cardiohelp^TM^ system has a massive drawback since, in this system, it is not possible to remove or exchange single system components such as the oxygenator [30]. Therefore, the Cardiohelp^TM^ system is more suitable for patients in which prompt RVAD weaning is more likely.

## 5. Conclusions

The study results suggest that the Deltastream^TM^ ECLS system significantly underperforms in intra-hospital mortality, which is most likely driven by bleeding complications and increased transfusions caused by the increase in thrombocyte destruction.

In the Deltastream^TM^ group, higher bleeding complications are likely attributable to the system’s elevated pump revolution rates, which increase shear stress on circulating blood elements, particularly thrombocytes. This mechanical stress may cause platelet activation, fragmentation, or destruction, leading to thrombocytopenia and increased bleeding risk. The need for higher anticoagulation to prevent thrombus formation in response to these higher revolution rates further compounds the bleeding risk. While additional data, such as platelet function assays, would strengthen this hypothesis, the current understanding of shear stress and platelet dysfunction provides a plausible explanation for the observed complications. Future studies should explore alternative pump settings or anticoagulation strategies to mitigate this risk. The Cardiohelp^TM^ group had a lower INTERMACS score, which supported referrals to our institution and is a good solution for initial patient support, even in high-risk patients. However, its design as a compact ECLS system without the possibility of removing an oxygenator limits its use for long-term support.

Considering all variables, our study showed that the Centrimag^TM^ system performed better regarding in-hospital mortality, reoperation due to bleeding, postoperative transfusion, and ICU stay duration. However, various factors, including patient characteristics and disease severity, can influence these outcomes. Therefore, the choice of ECLS system should be tailored to the individual patient’s needs and condition.

The retrospective and monocentric design of our study presents certain limitations that need to be acknowledged. Selection bias could have occurred since the choice of RVAD system was not standardised but rather based on availability at the time of implantation. This has led to imbalances in baseline characteristics between the groups, potentially affecting the outcomes. Additionally, being a retrospective study, the data were collected from medical records, which could result in incomplete or inconsistent documentation, leading to potential misclassification or inaccurate interpretation. Despite using multivariate analysis to adjust for baseline differences, unmeasured confounding factors such as preoperative hemodynamic status may have influenced patient outcomes. Furthermore, as this was a single-centre study, the findings may not be generalisable to other institutions with different practises.

To mitigate these limitations in future research, conducting randomised controlled trials across multiple centres would reduce selection bias and enhance the generalisability of the results. Implementing standardised protocols for device selection, anticoagulation management, and postoperative care would allow for a more direct comparison of outcomes. Additionally, increasing the sample size would enhance statistical power and provide more robust conclusions. In summary, while our study is limited by its retrospective and monocentric nature, these limitations can be addressed through carefully designed multicentre, prospective trials in future research.

Nevertheless, despite the highly challenging postoperative clinical course, more than a third of patients (38.0%) survived more than three years after the surgery, making temporary RVAD implantation still a viable solution for these terminally ill patients.

## Figures and Tables

**Table 1:** Preoperative and intraoperative patient characteristics.

	Centrimag^TM^ (*n* = 146)	Cardiohelp^TM^ (*n* = 46)	Deltastream^TM^ (*n* = 53)	*p*
Preoperative parameters				
Age (years)	52.7 ± 13.0; 55.5; 17	51.9 ± 11.0; 52.5; 12	56.5 ± 9.2; 66.0; 11 ^‡^	0.039
Body Mass Index	26.7 ± 5.4; 25.5; 6.2	27.3 ± 6.3; 25.4; 5.8	26.2 ± 5.1; 25.4; 6.9	0.926
Sex (male, n (%))	110 (75.3%)	38 (82.6%)	45 (84.9%)	0.269
INTERMACS Score	1.71 ± 0.75; 2; 1	1.26 ± 0.54; 1; 0 ^†^	1.91 ± 0.93; 2; 2 ^‡^	<0.001
LVEF (%)	20.0; 10.0	17.0; 10	20.0; 10.0	0.041
Previous Cardiac Surgery, n, (%)	25 (17.1%)	4 (8.7%)	16 (30.2%) ^†,‡^	0.009
Neurology, n, (%)	19 (13.0%)	6 (13.0%)	8 (15.1%)	0.926
Previous CVI, n (%)	16 (11.0%)	5 (10.9%)	8 (15.1%)	0.709
PAOD, n (%)	11 (7.5%)	2 (4.3%)	6 (11.3%)	0.428
Diabetes Mellitus, n (%)	45 (30.8%)	15 (32.6%)	12(22.6%)	0.464
Dialysis, n (%)	5 (3.4%)	0 (0.0%)	1 (1.9%)	0.405
Urgency, n (%)	119 (95.2%)	36 (100.0%)	44 (95.7%)	0.413
Pulmonary Hypertension, n (%)	82 (56.2%)	28 (60.9%)	35 (66.0%)	0.441
Preoperative CPR, n (%)	5 (3.4%)	7 (15.2%) ^†^	3 (5.7%)	0.014
Cardiomyopathies				
Ischemic Cardiomyopathy	54 (37.0%)	21 (45.7%)	30 (56.6%) ^†^	0.043
Dilated Cardiomyopathy	50 (34.3%)	17 (37.0%)	17 (32.1%)	0.261
Dilated Cardiomyopathy: Myocarditis	31 (21.2%)	3 (6.5%) ^†^	2 (3.8%) ^†^	0.002
Dilated Cardiomyopathy: Toxic	6 (4.1%)	2 (4.3%)	2 (3.8%)	0.989
Hypertrophic Cardiomyopathy	1 (0.7%)	0 (0.0%)	2 (3.8%)	0.999
Restrictive Cardiomyopathy	1 (0.7%)	1 (2.2%)	0 (0.0%)	0.999
Valvular Heart Disease	1 (0.7%)	2 (4.3%)	0 (0.0%)	0.999
Congenital Heart Disease	2 (1.4%)	0 (0.0%)	0 (0.0%)	0.999
Preoperative MCS				
Preoperative ECLS, n (%)	43 (29.5%)	26 (56.5%) ^†^	14 (26.4%) ^‡^	0.001
Preoperative IABP, n (%)	32 (21.9%)	8 (17.4%)	7 (13.2%)	0.364
Preoperative Impella^TM^, n (%)	9 (6.2%)	8 (17.4%) ^†^	3 (5.7%)	0.040
Intraoperative parameters				
Cardiopulmonary Bypass, n (%)	124 (84.9%)	27 (58.7%) ^†^	41 (77.4%)	<0.001
Hemofiltration, n (%)	62 (42.5%)	7 (15.2%) ^†^	19 (35.8%) ^‡^	0.004
Cell-Saver, n (%)	137 (93.8%)	42 (91.3%)	48 (90.6%)	0.689
LVAD Type				
HVAD^TM^, n (%)	105 (71.9%)	41 (89.1%) ^†^	40 (75.5%)	0.040
HM3^TM^, n (%)	41 (28.1%)	5 (10.9%) ^†^	13 (24.5%)	
Bypass Time (min)	138.4 ± 65.9; 125.5; 84	130.7 ± 72.5; 108; 72	143.9 ± 55.7; 135; 84	0.712
Body Temperature (°C)	33.6 ± 6.6; 35.0; 2.0	30.7 ± 12.1; 34.9; 1.8	31.6 ± 10.4; 34.6; 1.8	0.084
Oxygenator, n (%)	101 (69.2%)	46 (100.0%) ^†^	44 (83.0%) ^‡^	<0.001

INTERMACS Score—Interagency Registry for Mechanically Assisted Circulatory Support Score; LVEF—left ventricular ejection fraction; CVI—cerebrovascular insult; PAOD—peripheral artery disease, CPR—cardio pulmonary resuscitation; MCS—mechanical circulatory support; ECLS—extracorporeal life support; IABP—intra-aortic balloon pump; ^†^—*p* < 0.05 vs. Centrimag^TM^; ^‡^—*p* < 0.05 vs. Cardiohelp^TM^.

**Table 2:** Preoperative laboratory findings.

	Centrimag^TM^ (*n* = 146)	Cardiohelp^TM^ (*n* = 46)	Deltastream^TM^ (*n* = 53)	*p*
Creatinine (mg/dL)	1.60 ± 0.81; 1.4; 1.0	1.73 ± 0.99; 1.35; 1.49	1.76 ± 0.95; 1.5; 1.05	0.581
Urea (mg/dL)	78.8 ± 45.9; 66.5; 58	68.2 ± 47.4; 52.5; 68	72.3 ± 47.2; 59.0; 44	0.131
GFR (mL/min)	56.8 ± 28.0; 52.5; 39	59.7 ± 36.5; 52.0; 50	51.4 ± 26.0; 47.0; 36	0.535
Haemoglobin (g/dL)	10.7 ± 1.9; 10.2; 2.3	10.9 ± 1.8; 10.5; 1.9	10.2 ± 1.7; 9.9; 2.1	0.099
Plasma Free Haemoglobin (mg/dL)	12.8 ± 19.9; 8.0; 8	15.9 ± 17.9; 9.0; 11	12.2 ± 11.7; 9.0; 9	0.446
Haematocrit (%)	32.2 ± 5.5; 31.2; 7	32.5 ± 4.7; 30.9; 5	31.2 ± 5.1; 30.5; 7	0.314
Thrombocytes (10^9^/L)	159.4 ± 89.9; 144; 104	122.3 ± 72.8 ^†^; 109; 98	160.6 ± 103.6; 150; 116	0.030
CK (U/L)	670.9 ± 2447.0; 62.0; 184	1163.8 ± 3097.9; 197.0; 840 ^†^	473.7 ± 1198.9; 55.0; 330	0.010
CK MB (ng/mL)	17.9 ± 59.0; 2.3; 6.9	54.8 ± 206.9; 3.2; 10.7	9.85 ± 15.86; 3.0; 7.8	0.630
Bilirubin (mg/dL)	2.36 ± 2.03; 1.74; 1.6	2.75 ± 2.98; 1.73; 2.4	2.36 ± 2.35; 1.64; 1.5	0.821
ALT (U/L)	257.2 ± 614.4; 55; 121	529.1 ± 1105.4; 98.0; 375	140.7 ± 404.8; 29; 69 ^‡^	0.008
GGT (U/L)	167.1 ± 165.4; 113.5; 138	189.5 ± 189.4; 122.0; 180	238.9 ± 272.0; 132.0; 157	0.204
AST (U/L)	290.8 ± 844.4; 53.5; 90	1126.7 ± 2887.8; 76.0; 303 ^†^	213.5 ± 660.5; 41.0; 60 ^‡^	0.008
LDH (U/L)	693.0 ± 1170.3; 376; 272	726.2 ± 1170.4; 497; 785	726.2 ± 1170.4; 325; 288 ^‡^	0.003
Alkaline phosphatase (U/L)	121.3 ± 78.1; 106; 58	115.8 ± 64.4; 96; 77	137.5 ± 81.5; 122; 64	0.142
INR	1.61 ± 0.71; 1.30; 0.6	1.60 ± 0.73; 1.30; 0.8	1.31 ± 0.26; 1.20; 0.4	0.066
PTT (s)	45.6 ± 22.2; 41.0; 17	52.3 ± 29.6; 45.5; 19	44.3 ± 23.5; 38.0; 17	0.097
Fibrinogen (mg/dL)	356.7 ± 147.5; 334.0; 175	408.5 ± 197.1; 360.5; 243	360.5 ± 110.6; 370.0; 167	0.355
Antithrombin III (%)	76.1 ± 18.7; 74.0; 24	76.1 ± 23.8; 78.0; 26	84.0 ± 22.5; 82.0; 19	0.064

GFR—glomerular filtration rate; CK—creatin kinase; ALT—alanine aminotransferase; GGT—gamma-glutamyl transferase; AST—aspartate aminotransferase; LDH—lactate dehydrogenase; INR—international normalised ratio; PTT—partial thromboplastin time; ^†^—*p* < 0.05 vs. Centrimag^TM^; ^‡^—*p* < 0.05 vs. Cardiohelp^TM^.

**Table 3:** Study endpoints.

	Centrimag^TM^ (*n* = 146)	Cardiohelp^TM^ (*n* = 46)	Deltastream^TM^ (*n* = 53)	*p*
Primary endpoints				
In-hospital death	61 (41.8%)	15 (32.6%)	29 (54.7%) ^†,‡^	0.079
Reoperation due to Bleeding	32 (21.9%)	8 (17.4%)	25 (47.2%) ^†,‡^	0.001
Secondary endpoints				
ICU stay (days)	47.3 ± 41.4; 34.0; 48	54.3 ± 48.4; 41.0; 42	68.8 ± 70.5; 49.0; 70 ^†^	0.100
Invasive Respiration (hours)	694.9 ± 732.4; 499.4; 953	801.6 ± 758.5; 689.7; 1012	1069.1 ± 932.6; 743.4; 1295 ^†^	0.020
Hospital stay (days)	95.7 ± 68.8; 82.0; 83	97.4 ± 60.5; 83.5; 81	107.5 ± 75.2; 85.0; 82	0.495
RVAD duration (days)	27.4 ± 27.7; 22.0; 22	20.8 ± 12.6; 16.0; 17	30.6 ± 27.0; 25.0; 22	0.105
Successful RVAD weaning	41 (28.1%)	15 (32.6%)	7 (13.2%) ^†,‡^	0.052
Heart transplant	36 (24.7%)	12 (26.1%)	5 (9.4%) ^†,‡^	0.050

ICU—intensive care unit; RVAD—right ventricular assist device; ^†^—*p* < 0.05 vs. CentrimagTM; ^‡^—*p* < 0.05 vs. CardiohelpTM.

**Table 4:** Multivariate analysis of risk factors influencing intra-hospital mortality and reoperation due to bleeding.

	OR	95% CI	*p*
Reoperation due to bleeding			
Preoperative Antithrombin III	1.02	1.00 to 1.04	0.021
Deltastream^TM^	2.66	1.31 to 5.42	0.007
Intra-hospital mortality			
Age	1.06	1.03 to 1.09	<0.001
Previous Cardiac Surgery	2.19	1.04 to 4.64	0.040
Preoperative serum LDH	1.00	1.00 to 1.00	0.031
Deltastream^TM^	2.68	1.04 to 6.92	0.041

LDH—lactate dehydrogenase.

**Table 5:** Blood products transfusions.

	Centrimag^TM^ (*n* = 146)	Cardiohelp^TM^ (*n* = 46)	Deltastream^TM^ (*n* = 53)	*p*
EC total (Units)	71.3 ± 51.5; 62.5; 72	64.7 ± 40.7; 53.5; 54	85.6 ± 47.4; 85.0; 75	0.054
EC intraoperative (Units)	3.03 ± 2.83; 3.0; 5	2.50 ± 2.99; 2.0; 4	3.15 ± 2.65; 3.0; 6	0.291
EC postoperative (Units)	54.80 ± 48.18; 44.5; 60	46.46 ± 39.48; 32.5; 47	73.06 ± 47.30; 69.0; 73 ^†,‡^	0.005
TC total (Units)	19.97 ± 17.67; 15.0; 21	17.91 ± 15.36; 15.0; 13	32.28 ± 27.72; 25.0; 27 ^†,‡^	<0.001
TC postoperative (Units)	15.18 ± 17.36; 9.5; 19	11.50 ± 13.36; 9.0; 12	28.26 ± 27.29; 21.0; 23 ^†,‡^	<0.001
FFP total (Units)	35.24 ± 25.35; 32.0; 33	30.1 ± 17.1; 29.0; 22	39.9 ± 28.8; 32.0; 34	0.405
FFP postoperative (Units)	24.04 ± 22.64; 20.0; 31	16.54 ± 17.47; 8.0; 24	30.81 ± 26.71; 25.0; 23 ^†,‡^	0.008

EC—erythrocytes concentrate; TC—thrombocyte concentrate; FFP—fresh frozen plasma; ^†^—*p* < 0.05 vs. CentrimagTM; ^‡^—*p* < 0.05 vs. CardiohelpTM.

**Table 6:** Multivariate analysis of risk factors influencing postoperative transfusion rates.

Transfusions of	B	95% CI	Beta	*p*
Erythrocyte concentrates				
INTERMACS Score	8.05	−0.30 to 10.62	0.13	0.047
Oxygenator	18.78	3.94 to 33.62	0.17	0.013
Preoperative AST	0.005	0.001 to 0.009	0.15	0.027
Deltastream^TM^	14.70	−0.44 to 29.83	0.13	0.057
Cardiohelp^TM^	−14.96	−31.63 to 1.72	−0.13	0.078
Thrombocytes concentrates				
Age	0.24	0.04 to 0.45	0.14	0.021
Preoperative serum AST	0.002	0.001 to 0.004	0.19	0.004
Preoperative serum Antithrombin III	−0.14	−0.26 to −0.02	−0.14	0.026
Deltastream^TM^	12.99	6.73 to 19.261	0.26	0.000
Fresh Frozen Plasma				
Age	0.28	0.023 to 0.53	0.143	0.033
Oxygenator	8.21	1.03 to 15.38	0.147	0.025
Preoperative Serum LDH levels	0.003	0.001 to 0.004	0.228	0.000
Cardiohelp^TM^	−11.97	−19.70 to −4.24	−0.203	0.003

INTERMACS Score—Interagency Registry for Mechanically Assisted Circulatory Support Score; AST—aspartate aminotransferase; LDH—lactate dehydrogenase.

## Data Availability

The raw data supporting the conclusions of this article can be made available by the authors upon request, pending acceptance by the clinic and approval by the ethical board.
